# Supplementary material for: Localised dynactin protects growing microtubules to deliver oskar mRNA to the posterior cortex of the Drosophila oocyte
Source: eLife. 2017 Oct 16;6:e27237. doi: 10.7554/eLife.27237 (PMC5643094; doi:10.7554/eLife.27237)
Supplement: Transparent reporting form [file elife-27237-transrepform.pdf]

eLIFE
1st Floor
24 Hills Road
Cambridge CB2 1JP, UK
P 01223 855340
W elifesciences.org
T @elife

eLife's transparent reporting form

We encourage authors to provide detailed information *within their submission* to facilitate the interpretation and replication of experiments. If you have any questions, please contact us: editorial@elifesciences.org.

Sample-size estimation

- You should state whether an appropriate sample size was computed when the study was being designed
- You should state the statistical method of sample size computation and any required assumptions
- If no explicit power analysis was used, you should describe how you decided what sample (replicate) size (number) to use

Please outline where this information can be found within the submission (e.g., page numbers or figure legends), or explain why this information doesn't apply to your submission:

oskar mRNA tracking experiments (Figure 6, Table 1):
We analysed all *oskar* mRNA particles present in the movies. For the quantification of the average speeds we tried to track about 900 particles in wild-type conditions and about 300 particles in the mutant. For the calculation of the mobile fractions, we analysed all particles present in the movies in which there was a significant amount of mRNA particles away from the oocyte anterior. We were limited by the abundance of animals with the required genotype and by the number of visible mRNA particles. Tracking was performed in at least 4 oocytes (replicates) per genotype.

EB1 tracking experiments (Figures 7A, 7D and 8B):
We analysed all EB1 comets present in the movies. However we were limited by the abundance of animals with the required genotype and by the number of EB1 comets present at the posterior region. Tracking was performed in at least 5 (Figure 7) or 3 (Figure 8) oocytes (replicates) per genotype.

Replicates

- You should report how often each experiment was performed
- You should include a definition of biological versus technical replication
- The data obtained should be provided and sufficient information should be provided to indicate the number of independent biological and/or technical replicates
- If you encountered any outliers, you should describe how these were handled
- Criteria for exclusion/inclusion of data should be clearly stated
- High-throughput sequence data should be uploaded before submission, with a private link for reviewers provided (these are available from both GEO and ArrayExpress)

eLife Sciences Publications, Ltd is a limited liability non-profit non-stock corporation incorporated in the State of Delaware, USA, with company number 5030732, and is registered in the UK with company number FC030576 and branch number BR015634 at the address 1st Floor, 24 Hills Road, Cambridge CB2 1JP | August 2014

Please outline where this information can be found within the submission (e.g., page numbers or figure legends), or explain why this information doesn't apply to your submission:

For the quantitative analysis experiments, the sample number and replicates are indicated in the respective figure legend, table or Material and Method section.
In live imaging experiments, samples were always excluded from the analysis when there were doubts about the oocyte viability or because of poor quality microscopy.

Statistical reporting

- Statistical analysis methods should be described and justified
- Raw data should be presented in figures whenever informative to do so (typically when N per group is less than 10)
- For each experiment, you should identify the statistical tests used, exact values of N, definitions of center, methods of multiple test correction, and dispersion and precision measures (e.g., mean, median, SD, SEM, confidence intervals; and, for the major substantive results, a measure of effect size (e.g., Pearson's r, Cohen's d)
- Report exact p-values wherever possible alongside the summary statistics and 95% confidence intervals. These should be reported for all key questions and not only when the p-value is less than 0.05.

Please outline where this information can be found within the submission (e.g., page numbers or figure legends), or explain why this information doesn't apply to your submission:

The statistical methods used are described in the Material and Methods section, as well as in the figure legends. The exact P values are stated in the figure legends.

(For large datasets, or papers with a very large number of statistical tests, you may upload a single table file with tests, Ns, etc., with reference to page numbers in the manuscript.)

Additional data files ("source data")

- We encourage you to upload relevant additional data files, such as numerical data that are represented as a graph in a figure, or as a summary table
- Where provided, these should be in the most useful format, and they can be uploaded as "Source data" files linked to a main figure or table
- Include model definition files including the full list of parameters used
- Include code used for data analysis (e.g., R, MatLab)
- Avoid stating that data files are "available upon request"

eLIFE
1st Floor
24 Hills Road
Cambridge CB2 1JP, UK
P 01223 855340
W elifesciences.org
T @elife

Please indicate the figures or tables for which source data files have been provided:

Table 1: Table 1_source data1, Table 1_source data2, Table 1_source data3
Figure 6A: Figure 6_source data1
Figure 6B: Figure 6_source data2
Figure 7A: Figure 7_source data1
Figure 7D: Figure 7_source data2
Figure 7E: Figure 7_source data3
Figure 8B: Figure 8_source data1

eLife Sciences Publications, Ltd is a limited liability non-profit non-stock corporation incorporated in the State of Delaware, USA, with company number 5030732, and is registered in the UK with company number FC030576 and branch number BR015634 at the address 1st Floor, 24 Hills Road, Cambridge CB2 1JP | August 2014
